# New Insight on Promoted thermostability of poplar wood modified by MnFe_2_O_4_ nanoparticles through the pyrolysis behaviors and kinetic study

**DOI:** 10.1038/s41598-017-01597-4

**Published:** 2017-05-03

**Authors:** Hanwei Wang, Qiufang Yao, Chao Wang, Bitao Fan, Ye Xiong, Yipeng Chen, Qingfeng Sun, Chunde Jin, Zhongqing Ma

**Affiliations:** 1 School of Engineering, Zhejiang A&F University, Lin’an, 311300 P.R. China; 2 Key Laboratory of Wood Science and Technology, Zhejiang Province, 311300 P.R. China

## Abstract

In this study, we employed pyrolysis behavior and kinetics by Flynn–Wall–Ozawa method and Friedman method to analysis the thermostability of the MnFe_2_O_4_ nanoparticles/poplar wood composite, and analyzed the change of different proportion of MnFe_2_O_4_ in these composites for the thermostability by contrasting activation energy between the different samples. The pyrolysis processes of these composites were comprehensively investigated at different heating rates (10, 20, 30 and 40 °C/min^−1^) and pyrolysis temperatures of 600 °C in N_2_ and air atmosphere. These results indicated the thermostability of composites improved as the proportion of the MnFe_2_O_4_ nanoparticles increased. And the structure analyses of these composites from the microscopic view point of nanoparticles were applied to analysis the reason of thermostability enhancement of the poplar wood after coating MnFe_2_O_4_ nanoparticles. Additionally, due to its high initial oxidative decomposition temperature under air atmosphere, this composite and its preparation method might have high application potential, such as flameresistant material and wood security storage. This method also could provide a reference for other biomass materials. Synthesized MnFe_2_O_4_/C composite under the guidance of pyrolysis behaviors and kinetic study in N_2_ atmosphere exhibited good adsorption capacity (84.18 mg/g) for removing methylene blue dye in aqueous solution and easy separation characteristic.

## Introduction

The fast-growing poplar, as a typical biomass material, received a great attention by the advantages of high-yielding for these short rotation periods and widely plant, applied in the fields of building materials, artificial board and so on refs 1 and 2. However, low thermostability performance of poplar wood by nature limited its widely application. Recently, many researchers showed coated the inorganic nanoparticles on the wood surface, such as Na_2_O-SiO_2_^3^, ZnO^4^ and Al(OH)_3_^5^. These methods could not only endow the wood with novel functional properties, but also improved the thermostability properties of composites. The burning method, as an important measurement for thermostability, was usually used to estimate the thermostability and flame retardant performance for the wood composite samples^4, 6^. Nevertheless, the environmental problem and artificial error from artificial observation made this method not very suitable to determine the thermostability for these samples. Thus, due to features high accuracy, good reproducibility, smaller sample consumption, fast measurement and simple operation, the thermogravimetric analysis under the N_2_ and air atmosphere was widely applied in determination of the thermostability of samples. But this method in many studies was just a simple analysis of the TG and DTG curves for detection of the thermostability^7, 8^.

The pyrolysis behaviors study was generally on the base of the thermogravimetric analysis^9, 10^. The non-isothermal kinetics as a thermal analysis kinetic was a key method to analysis the pyrolysis process due to that could calculate the kinetic parameters continuous during the whole pyrolysis process^11, 12^. In this kinetics, the activation energy of samples could be easily calculated. These activation energies could represent the minimum energy to trigger a chemical reaction^13^. In other words, to trigger a difficult chemical reaction needed higher activation energy. It means that the activation energy during the whole pyrolysis process in the different samples could relatively represent their thermostability at the same experimental conditions and kinetic model. Thus, calculated activation energy from the whole pyrolysis process under N_2_ and air atmosphere was useful for assessing thermostability of samples. Additionally, pyrolysis behaviors study under N_2_ atmosphere also could provide a technology for the preparation of inorganic/C composite based on the study of the pyrolysis process of inorganic/wood composite.

The model-free methods apply multiple TGA curves of the same sample at the different heating rates to analysis the thermal kinetic, and have good accuracy compared the model-fitting methods that only used one TGA curve in the single heating rate^14–18^. In this method, the assumption that the change in reaction rate is proportional to the rate constant alone for this method is essential^19^. In other wood, this means that the reaction rate depends only on the temperature in a constant extent of conversion. Currently, to determine the pyrolysis behaviors and kinetics, the model-free methods were used by various researches in the different kinds of biomass, such as palm kernel, poplar wood, bamboo and so on refs 13, 20 and 21. However, few studies applied the pyrolysis behavior and kinetics to research the thermostability for poplar wood.

The previous study had indicated that the MnFe_2_O_4_ nanoparticles/poplar wood composite have excellent performance of the superparamagnetic and microwave absorption performance, and the MnFe_2_O_4_ nanoparticles have favorable chemical stability and play an important role for protection of the wood substrate^22^. In this work, we further investigated thermostability of the MnFe_2_O_4_/wood composite by TG/DTG and DSC analysis under the N_2_ and air atmosphere. The corresponding activation energies, as an important assessment method for thermostability, were calculated by the Flynn–Wall–Ozawa (FWO) integral method and Friedman differential methods with the different conversion rate (α) and multi-heating rate method (heating rates of 10, 20, 30, and 40 °C min^−1^)^23–25^. The obtained results would be compared to the validity and performance of the models for the experimental. Then, the physics and chemical structure of these composite were further analyzed to investigate the reasons for change of thermostability by the X-ray diffraction technique (XRD), Transmission electron microscope (TEM), Fourier transform infrared spectroscopy (FT-IR) and X-ray photoelectron spectroscopy (XPS) analysis. And in order to obtain effect results for the different proportion of MnFe_2_O_4_ that influenced the thermostability of initial composite, these samples were prepared with different contents gradient for MnFe_2_O_4_ nanoparticles. And the MnFe_2_O_4_/wood composite represented high initial oxidative decomposition temperature in air atmosphere, implying wide applications for this composite in flameresistant material and security storage of wood. This method that coating MnFe_2_O_4_ nanoparticles to surface of other biomass material through a simple and low temperature hydrothermal reaction also might have large application value for security storage of others biomass material. Additionally, the pyrolysis behavior for initial MnFe_2_O_4_/wood composite in nitrogen atmosphere was able to provide a theoretical guidance for the preparation of MnFe_2_O_4_/C composite. Obtained MnFe_2_O_4_/C composites due to its easy separation property in aqueous solution based on its excellent magnetic responsiveness were further applied in absorption of methylene blue.

## Results and Discussion

**Figure 1:**
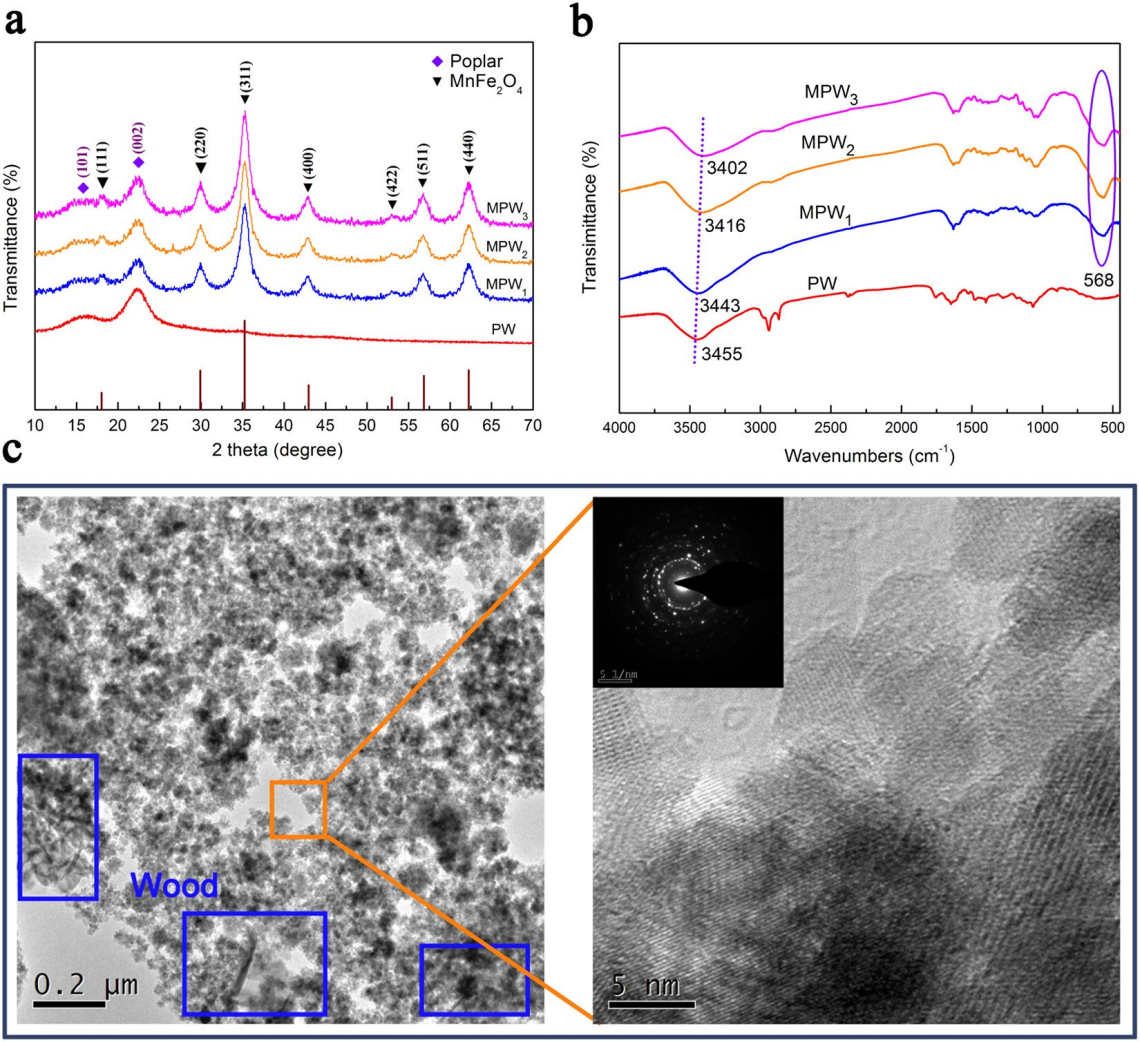
(**a**) XRD patterns and (**b**) FT-IR spectra of PW, MPW_1_, MPW_2_ and MPW_3_; (**c**) TEM images of MPW_2_.

The X-ray diffraction (XRD) was employed to analyze crystalline structures of the samples of PW, MPW_1_, MPW_2_ and MPW_3_ in Fig. 1a. All of the samples had two same strong diffraction peaks at 16.1° and 22.6°, indicating that the structures of poplar wood within the MPW composite still maintain after hydrothermal reaction^26^. The other diffraction peaks at 17.8°, 30.2°, 35.3°, 43.2°, 53.5°, 57.1° and 62.7° confirm the presence of the spinel MnFe_2_O_4_ (JCPDS 73-1964)^22^. Among them, the MPW_1_, MPW_2_, and MPW_3_ showed nearly the XRD patterns, indicating that these samples have the same crystalline structures. These results might imply that an important variable for different of the crystalline structures of the samples were excluded. In addition, the TEM images in Fig. 1c indicated that the MnFe_2_O_4_ nanoparticles have closely combined with wood surface.

The FT-IR analysis allowed further identification of the functional group from the PW and MPW. Figure 1b showed FT-IR spectra of PW, MPW_1_, MPW_2_ and MPW_3_. As shown in Fig. S3, excepting for the PW, all samples appeared a strong peak at 568 cm^−1^ attributed to the intrinsic vibrations of the manganese ferrite (Fe-O and Mn-O), which indicated the presence of MnFe_2_O_4_^27^. In addition, the peaks at 3500–3400 cm^−1^ were attributed to the –OH stretching absorption banding arising from hydroxyl groups from wood or nanoparticle surface^28^. In particular, these bands assigned for –OH group were shifted to lower wavenumbers with increment of the MnFe_2_O_4_ nanoparticles proportion, ascribing to the increased hydrogen bonds happened between the hydroxyl groups of the wood surface and the MnFe_2_O_4_ nanoparticles by the cross-linking reaction^29^.

**Figure 2:**
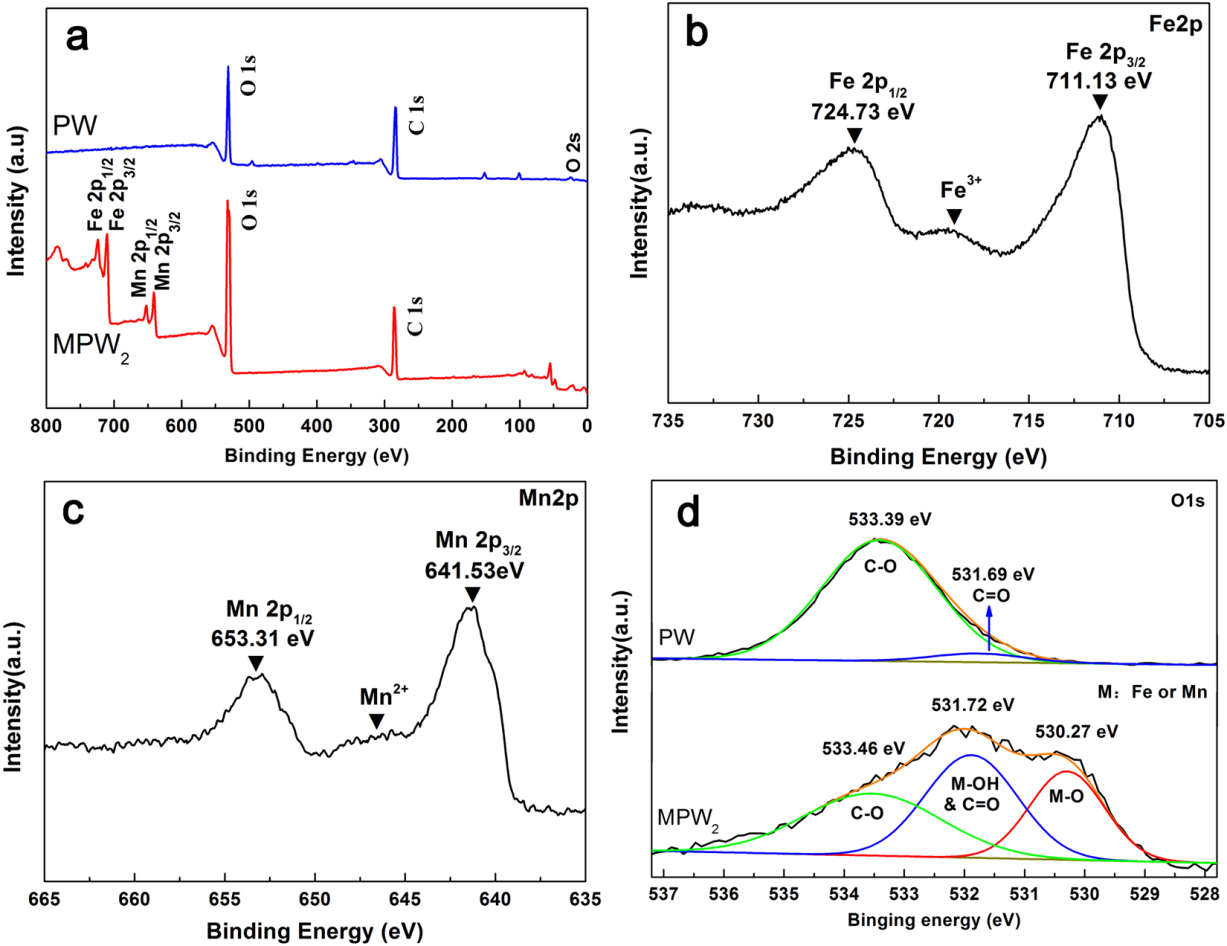
(**a**) Survey-scan XPS spectra of the PW and MPW_2_, (**b**) Fe2p spectra of the MPW_2_, (**c**) Mn2P spectra of the MPW_2_, and (**d**) O1s spectra of the PW and MPW_2_.

The XPS analysis provided the main composition elements and chemistry bonds of PW and MPW_2_. As shown in Fig. 2a, MPW_2_ exhibited the existence of Mn and Fe elements, but the PW did not have. And the Fe and Mn spectra could prove the presence of Fe^3+^ and Mn^2+^ ions in Fig. 2a,b ^30^. Then, the peak at 531.72 eV in the O1s spectra was attributed to carbon-oxygen double bond (such as lignin) and the –OH group from the surface of the MnFe_2_O_4_ nanoparticles, which offered a further evidence for the formation of hydrogen bonds^31^. In addition, the MPW_2_ spectra appeared the peak of Mn-O and Fe-O bonds at 530.27 eV. In summary, these results indicated the MnFe_2_O_4_ had successful united with the poplar wood by hydrogen bonds via a low-temperature hydrothermal reaction.

**Figure 3:**
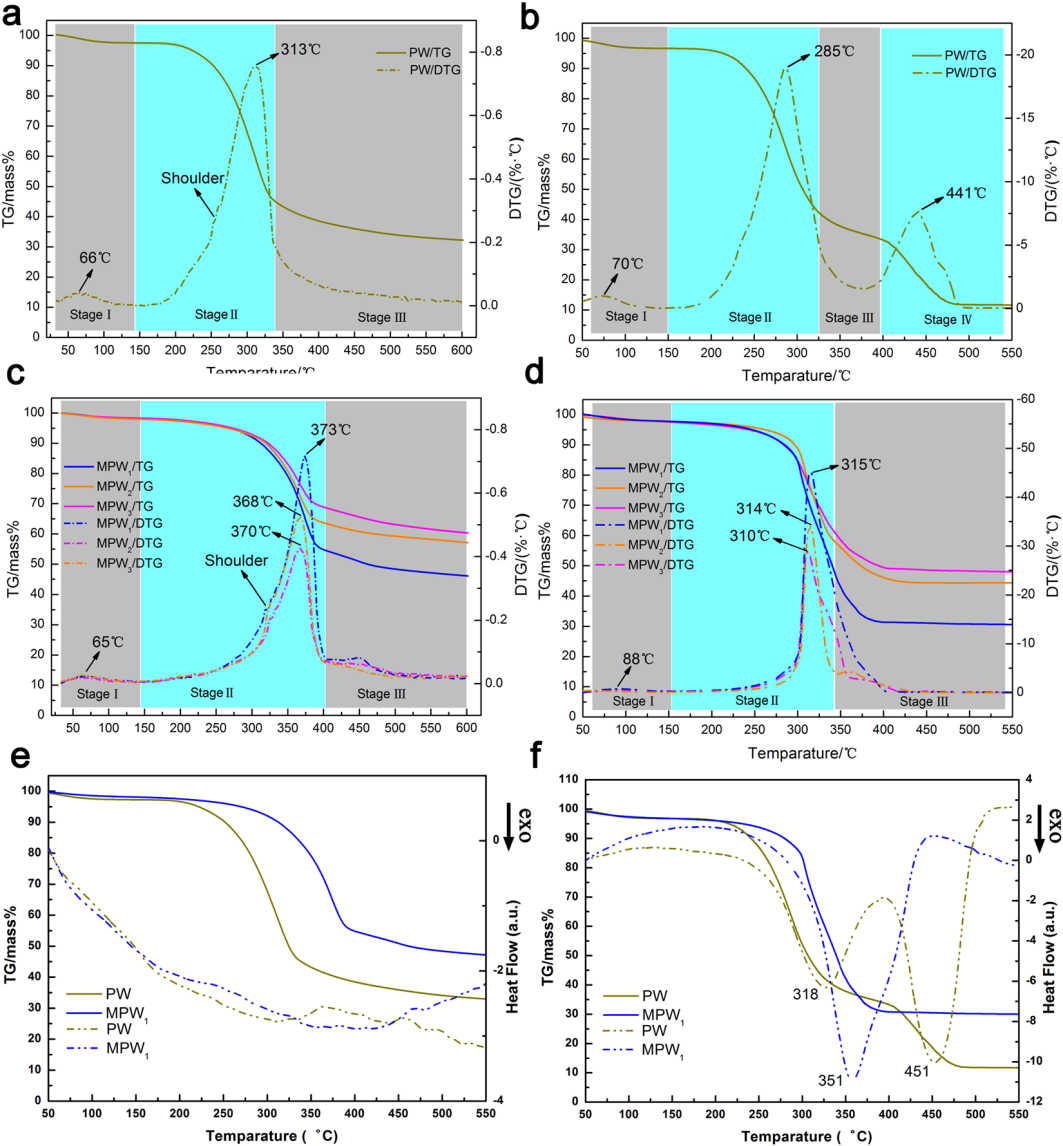
TG/DTG curves at heating rate of 20 °C/min of (**a**,**b**) PW and (**c**,**d**) MPW in nitrogen and air atmosphere, respectively. TG/DSC curves at heating rate of 20 °C/min of PW and MPW_1_ in (**e**) nitrogen and (**f**) air atmosphere.

TG/DTG experiments of PW, MPW_1_, MPW_2_ and MPW_3_ were performed under heating rate (20 °C/min) and the corresponding curves were exhibited in Fig. 3a,c. And the thermal decomposition of these samples from the temperature of 35 °C to 600 °C in a nitrogenous atmosphere could be divided into three stages in the TG and DTG curves. More importantly, compared with the TG curves of the PW, these curves in the MPW were excellent agreement with each other. That indicated the MnFe_2_O_4_ was a stable ingredient in this composite. Thus, the pyrolysis process of the MPW could be regarded as the degradation of the poplar wood.

As shown in Fig. 3a,c the first stage of the PW and PMW went from ambient temperature to about 143 °C. A little weight loss (PW of 2.49%, MPW_1_ of 1.94%, MPW_2_ of 2.04% and MPW_3_ of 1.69%) in the TG curve and a corresponded peak (PW of 66 °C and MPW_1_, MPW_2_ and MPW_3_ of 65 °C) in the DTG curve were observed, attributing to the mass loss of sample for the evaporation of adsorbed water and light volatiles component.

The second stage (from 143 °C to 338 °C for PW, from 143 °C to 403 °C for MPW_1_, MPW_2_ and MPW_3_) was the main process of pyrolysis with high mass loss proportion (PW of 52.34%, MPW_1_ of 43.45%, MPW_2_ of 34.39% and MPW_3_ of 29.61%) caused by the fast devolatilization of the wood. In this stage, large amounts of compound of small molecular weight, such as H_2_O, CO and CO_2_, might be produced during the decomposition process for poplar wood due to its large number of hydroxyl groups and oxygen atoms from the cellulose, hemicellulose and lignin^32^. One little shoulder peak appearing in the DTG curve was due to the peaks overlaps for the decomposition of hemicellulose. And the main peak, corresponding to the pyrolysis of cellulose, was also observed in the DTG curve represented the maximum decomposition rate of the cellulose^3, 9^. The shoulder peak and the main peak were appearing at 256 °C and 313 °C in the PW curve, respectively. Compared with the PW, this shoulder peak and main peak in the MPW_1_, MPW_2_ and MPW_3_ were separately shifting to higher temperatures, indicating that the thermostability of the MPW enhanced greatly after coating MnFe_2_O_4_ nanoparticles to the poplar wood surface on the hydrothermal process. Then, the TG and DTG curves of MPW_1_, MPW_2_ and MPW_3_ were in broad agreement with each other, and had the same position for this shoulder peak and main peak at about 323 °C and 370 °C as shown in Fig. 3c. In addition, the maximum decomposition rate of these samples decreased gradually with the increment of the MnFe_2_O_4_ nanoparticles proportion, which could be attributed to the proportion of wood decreased.

The third stage as a slowly degradation stage (PW of 338 °C–600 °C, MPW_1_, MPW_2_ and MPW_3_ of 403 °C–600 °C) could be ascribed to the degradation of the lignin. Except continuous produced H_2_O, CO and CO_2_ in this process for poplar wood, CH_4_ was also produced from decomposition of lignin side groups and as a result of charring processes^32^. In this stage, a small part of the weight of the PW, MPW_1_, MPW_2_ and MPW_3_ in the TG curves had lost corresponding to 12.72%, 8.5%, 6.45% and 8.39%, respectively. After the TG experiment, because of the different proportion of MnFe_2_O_4_ in these samples, the corresponding residue mass of these samples separately maintained at 32.45%, 46.11%, 57.12% and 60.31%. Thus, the thermostability of the poplar wood had increased by combining with the magnetic MnFe_2_O_4_ nanoparticles on the poplar wood surface. The mass percentage of residue left from the wood in the final decomposition product could be estimate by calculated theoretical content of MnFe_2_O_4_ within the composites. Thus, the residues of the MPW_1_, MPW_2_ and MPW_3_ from wood were 30.51%, 30.16%, and 28.74%, respectively. This result was close to the value of residue left of wood.

**Figure 4:**
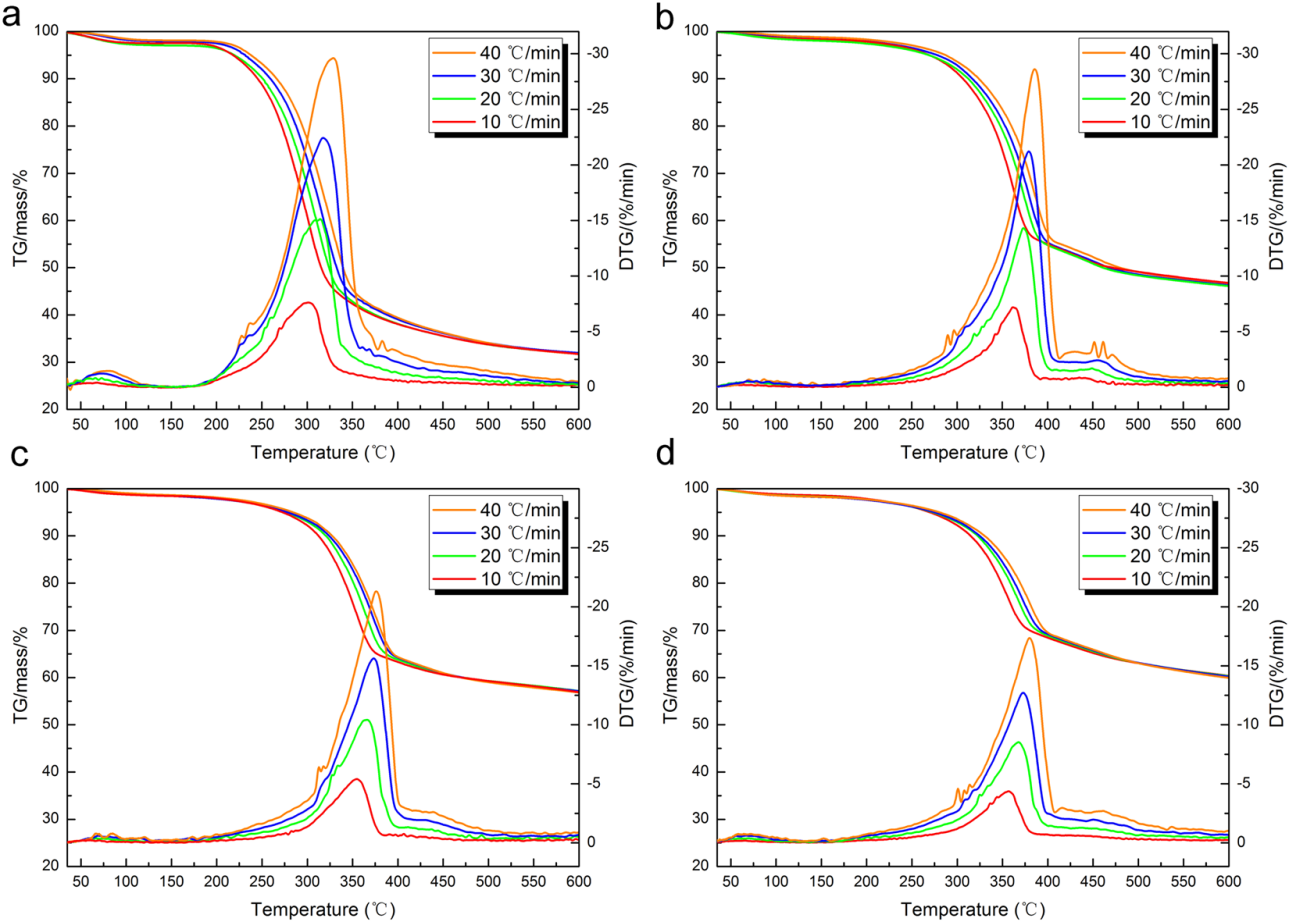
TG and DTG curves of (**a**) PW, (**b**) MPW_1_, (**c**) MPW_2_ and (**d**) MPW_3_ with heating rates of 10, 20, 30 and 40 °C/min in nitrogen atmosphere.

Figure 4a–d showed the temperature ranges of the pyrolysis process in the TG and DTG curves were influenced by the different heating rate (10, 20, 30 and 40 °C/min) using multi-heating rate method in N_2_ atmosphere. The onset of the devolatilization and peak temperatures in the DTG curves for all samples were shifted to higher temperatures, which could be attributed to the heat and mass transfer limitations of the samples^15^. And, furthermore, because of the biomass materials or these composites with a poor thermal conductivity, the temperature gradient was generated between surface and core of the sample particle. In addition, the thermal degradation rates of the samples in the DTG curves increased with the heating rate. Table 1 showed the experimental data for the pyrolysis process of the PW, MPW_1_, MPW_2_ and MPW_3_. As shown in Table 1, the yield of the residue materials reduced with the heating rate. It was generally believed that the viscosity of the melted solid material, in the process of intensifying pyrolysis, was reduced in the higher heating regime due to the heat fluxes increased with increasing of heating regime^33^.

**Table 1:** Effect of heating rate on TGA pyrolysis for the PW, MPW_1_, MPW_2_ and MPW_3_ in nitrogen atmosphere.

Sample	β (°C/min)	r_m_ (wt.%/min^−1^)	T_m_ (°C)	Residue (%dry)
PW	10	−7.61	302.28	31.97
	20	−15.13	314.57	31.80
	30	−22.43	317.86	31.73
	40	−29.34	331.79	31.70
MPW_1_	10	−7.18	361.05	46.78
	20	−14.26	372.44	46.71
	30	−21.21	380.22	46.37
	40	−28.61	385.85	46.12
MPW_2_	10	−5.40	355.69	57.17
	20	−10.41	365.61	57.15
	30	−15.63	375.92	56.92
	40	−21.31	376.15	56.85
MPW_3_	10	−4.37	357.55	60.38
	20	−8.53	369.06	60.33
	30	−12.71	373.47	60.21
	40	−17.34	379.34	59.96

**Figure 5:**
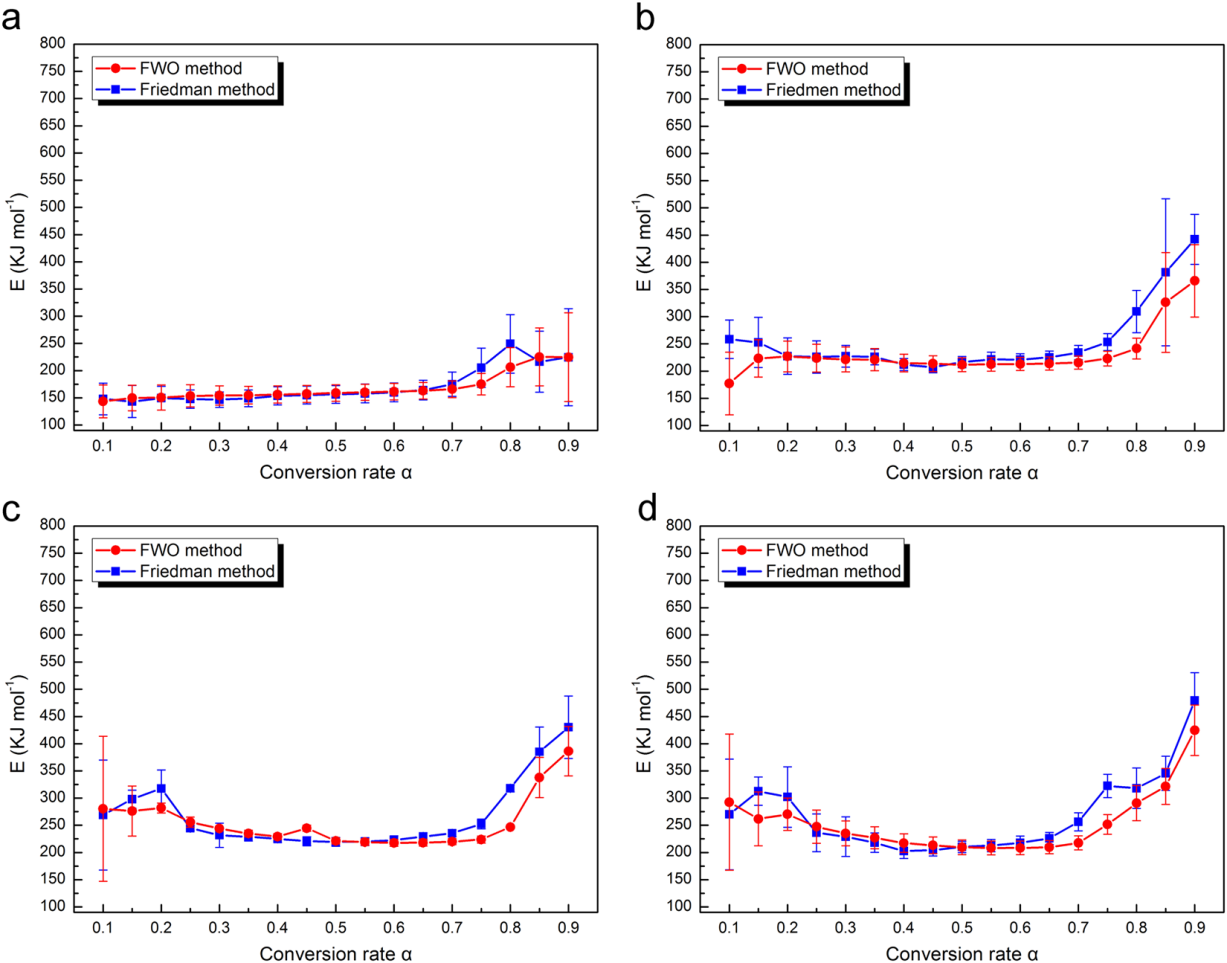
Activation energy (E) distribution with corresponding error bar in the different conversion rates using the FWO and Friedman methods for (**a**) PW, (**b**) MPW_1_, (**c**) MPW_2_ and (**d**) MPW_3_ in nitrogen atmosphere.

Analysis in terms of the activation energy of the PW, MPW_1_, MPW_2_ and MPW_3_ was presented in Fig. 5a,b,c and d, respectively. That revealed the typical behavior of the complex reactions, including parallel, multiple and consecutives during the degradation of the poplar wood and its composites in the nitrogen atmosphere^34, 35^.

Table S1 showed the values of the activation energies (E) and standard deviation at the different conversion values for the TG pyrolysis of the PW, MPW_1_, MPW_2_ and MPW_3_. These indicated that the estimated activation energy obtained by both free models kietics algorithms for FWO and Friedman methods had excellent agreement. This agreement of the results validated the reliability of the performed calculations and confirmed the excellent predictive power of the direct methods. And the multi-heating heating rate in the thermogravimetric experiment also improved the accuracy of the estimated activation energy^34^.

It was also worth mentioning that the activation energy could represent the minimum energy to trigger a chemical reaction. In other words, this means that to trigger a difficult chemical reaction needed the higher activation energy. In addition, several factors, such as heating rate, different kinetics model and samples, particle size and different types of TGA, could affect the value of activation energy. Therefore, the activation energy of these samples was only valid on the condition of given experiment parameters.

To obtain significant date and make effective contrast in all of the samples, it was necessary to use the available experimental date. Thus, the values of conversion were only above 0.1 and below 0.9 in these samples. As shown in Fig. 5, two trends of activation energy could be identified for all samples: 1) From values of the conversion 0.1 to 0.7, this could be mainly attributed to thermal degradation of the hemicellulose and cellulose. 2) For the values of the conversion at from 0.7 to 0.9, that could be attributed to the lignin degradation.

In particular, in order to more clearly describe the relationship between these samples, the FWO method was selected from the two methods. As it could be easily observed the contiguous stage between 0.1 and 0.7 for the conversion rate, showed a progressive increasing from 143 kJ/mol to 166 kJ/mol (FWO method) for the activation energy values in Fig. 5a. This result indicated the consecutive and competitive character of the processes during this stage, and the process of cross-linked polymer degradation could influence to the behavior of activation energy^6^. First, the broken of weakly linked site in the hemicellulose molecular chain provided the low activation energy at the initial of the decomposition process^28^. Then, as the reaction progress, the chemical bonds on the lineal chain of the hemicellulose gradually cleavage made the activation energy increased^36^. Same as the degradation process of the hemicellulose, the gradually pyrolysis of cellulose also led to higher activation energy, while the hemicellulose degradation reaction to a certain degree^10, 36^.

For the final trend (0.7 < α < 0.9), the activation energy fast increased with the temperature increased, which ranged from 166 kJ/mol to 225 kJ/mol (FWO method), which could be ascribed to the lignin degradation. And the heavily cross-linked three-dimensional network structure of lignin was mainly composed of three kinds of benzene-propane^36^. This caused that the degradation of lignin required highest energy by its distinctive structure in the complete experiments, and the main process of the decomposition of the lignin occurred around 400 °C. In addition, low reactivity of residual was the largest part of the sample during the last stage of process, which could explain the particular shape of the activation energy profile for the activation energy increased fast.

In general, the activation energy (0.1 < α < 0.9) ranged from 143 kJ/mol to 225 kJ/mol for PW, from 177 kJ/mol to 366 kJ/mol for MPW_1_, 280 kJ/mol to 386 kJ/mol for MPW_2_, and 292 kJ/mol to 425 kJ/mol for MPW_3_ (FWO method), indicating that the reaction activity of the sample reduced by increasing the content of MnFe_2_O_4_ in the samples. Compared with the activation energy profile of the PW, the MPW (all of the MPW samples) represented the higher activation energy in the whole decomposition reaction. These might due to the existence of the hydrogen bonds between the poplar wood and the MnFe_2_O_4_ (FTIR: Fig. 1b; XPS: Fig. 2), and the distinctive structure of the samples that the MnFe_2_O_4_ nanoparticles grown in surface of the wood particles and wrapped around it (SEM: Fig. 1c). In particular, the values of activation energy of the MPW had a trend of rise first then fall in the conversion rate from 0.1 to 0.4. These could be related to break of its distinctive structure that led to the wood particles bared gradually, and reduced the reaction activation energy.

In order to further investigate thermostability under the air atmosphere, TG/DTG curves for these samples were obtained by TG/DTG experiments at the different heating rate (10, 20, 30 and 40 °C/min). As shown in Fig. 3b, TG/DTG curve of wood showed four stages of the thermal degradation at the heating rate of 20 °C/min. Due to dehydration of wood and light volatiles component evaporation, there had a little mass loss of 3.21% in the first stage (50–150 °C). For the second stage, a 54.36% rapid mass loss occurred in the temperature range from 150 to 325 °C and a largest peak at 285 °C was also observed in the DTG curve, which could be attributed to oxidative decomposition of wood (hemicellulose, cellulose and lignin) where large amount of flammable volatile products were formed and then oxidative decomposition^37^. A slow mass loss (11.12%) of the residual materials of wood in third stage (325–400 °C) was found after oxidative decomposition process. In this stage, a highly condensed and cross-linked carbonaceous was gradually formed from the decomposition product of less stable aliphatic groups preferentially by the hemolytic cleavage of C-C and C-H bonds^38^. For the fourth stage, a mass loss of 21.14% was shown in TG curve in the temperature range 400–550 °C. And corresponding DTG curve also displayed a large peak at 441 °C. This mass loss could be attributed to the oxidation of residual carbonaceous.

TG/DTG curves of the MPW with different MnFe_2_O_4_ proportions at the heating rate of 20 °C/min showed three stages in Fig. 3d. Same as the PW, a little mass loss (MPW_1_ of 3.32%, MPW_2_ of 2.32% and MPW_3_ of 2.43%) appeared in the TG curves in first stage (50–150 °C). Interestingly, a large mass loss of TG curves for these composites began in a high temperature of about 250 °C, more than initial oxidative decomposition temperature of 150 °C for PW. The DTG curves of the composites also provided a shifted peak at a higher temperature of about 310 °C to prove promotion of the thermostability by coating MnFe_2_O_4_ to wood surface. These results suggested that coated MnFe_2_O_4_ nanoparticles represented inhibitory action for the oxidative decomposition of wood and delayed the temperature of initial reaction. Thus, the promotion of initial decomposition for wood implied good thermostability for the MPW before temperature of 250 °C in air^39^. Then, a fast weight loss and sharp peak were observed in TG and DTG curves after the temperature of 300 °C, implying a rapid manufacture of MnFe_2_O_4_/ember residues. In third stage (about 340–600 °C), a large mass loss of TG curves for MPW were observed in the temperature range from about 340 to 400 °C, ascribing to quick disappearing of flammability carbonaceous. This fast oxidative decomposition process for MPW in the short temperatures range from 300 to 400 °C provided high proportion of MnFe_2_O_4_/ember residues rapidly that might be able to resistance flame spread as a stable non-combustible material covered on the wood surface. At last, the weight of the MPW_1_, MPW_2_, and MPW_3_ residues maintained at 30.77%, 44.31%, and 48.29%, respectively. Thus, excepting for being used as a flame retardant material for MPW, these results also implied this method for coating MnFe_2_O_4_ to the surface of wood or other biomass material via a simple and low temperature hydrothermal reaction would have some potential applications in storage security of biomass material in long-term duration.

Figure S2a–d showed the TG/DTG curves for the temperature ranges of the pyrolysis process were influenced by the different heating rates (10, 20, 30 and 40 °C/min) using multi-heating rate method in air atmosphere. All of the MPW represented a high consistency in whole pyrolysis process. The onset and final of the oxidative decomposition and peak temperatures in DTG curves for all samples were shifted to higher temperatures, also attributing to the heat and mass transfer limitations of the samples. In particular, the TG/DTG for the same MPW sample in air atmosphere represented the obvious change at different heating rates. With the increase of the heating rate, growth of half peak width for DTG curves at high temperature side was much wider than the low temperature side due to heat and mass transfer limitations for oxidative decomposition of wood and carbonaceous^40^. This result indicated large numbers of nonflammable MnFe_2_O_4_/ember residues could be rapidly formed in the short range of temperatures that might be able to resistance flame spread. Table S2 showed the experimental data for the pyrolysis process of the PW, MPW_1_, MPW_2_ and MPW_3_.

As shown in Fig. S3a, two trends of activation energy could be identified for wood and MPW: 1) From values of the conversion 0.1 to 0.6, attributing to decomposition of wood (hemicellulose, cellulose and lignin). 2) For the values of the conversion at from 0.6 to 0.9, ascribing to oxidative decomposition of carbonaceous. Table S3 showed the values of the activation energies and standard deviation at the different conversion values for the PW, MPW_1_, MPW_2_ and MPW_3_ in air atmosphere. From Table S3 it was observed that for the low conversion values from 0.1 to 0.3, the MPW revealed higher activation energy (about 250 kJ/mol) compared with the PW (about 200 kJ/mol), indicating MnFe_2_O_4_ nanoparticles might have inhibitory action for initial oxidative decomposition of wood, which was also proved by its improved initial decomposition temperature in TG/DTG curves. This result might be attributed to synergistic effect of hydrogen bonds and coating structure in the MPW composites. Then, after the values of the conversion of 0.3, low activation energies for MPW and corresponding fast mass loss of TG curves indicated the rapid formation of MnFe_2_O_4_/ember residues in air atmosphere. Compared with the single ember from the PW, this high proportion of residue still remained high hardness and density that might be able to help slow the spread of flames. Additionally, the activation energy distribution curves for MPW in N_2_ and air atmosphere reflected an increase in overall with the increasing percent of the MnFe_2_O_4_, implying that added the proportion of MnFe_2_O_4_ within the composite could promote the thermostability of materials.

Finally, the thermal behaviors of the poplar wood and MnFe_2_O_4_/wood composite under N_2_ and air atmosphere were studied to establish the effect of MnFe_2_O_4_ for composite. For the attainment of this aim, the DSC curves of PW and MPW were carried out both in N_2_ and air streams, only the DSC curves of PW and MPW_1_ in Fig. 3c,d, as an example. As shown in Fig. 3c, continuous exothermic process was observed for PW and MPW_1_ in N_2_ streams. The pyrolysis temperature of the MPW_1_ at the pyrolysis process of hemicellulose and cellulose increased under the condition of coated MnFe_2_O_4_ nanoparticles according to the exothermic peak raised from about 300 °C to 380 °C in DSC curves. After this stage, the MPW_1_ became more stable as temperature rise. Under the air streams, two consecutive decomposition steps for the poplar wood were observed in the DSC curves, which could be attributed to the oxidative decomposition of the wood and carbon left, respectively^41^. The decomposition enthalpy of MPW_1_ was quite lower than that of the PW before the temperature of the 320 °C, and the first exothermic peak was also shifted to the higher temperature, implying the inhibitory action of coated MnFe_2_O_4_ nanoparticles for the oxidative decomposition of wood. The shoulder exothermic peak for MPW_1_ appeared at the temperature of 390 °C due to oxidative decomposition of carbon, which also could represented the rapid formation of high proportion and stable MnFe_2_O_4_/ember residues. However, compared with air atmosphere, these samples showed approximate trend for exothermic curves in N_2_ atmosphere with weak exothermic peaks that was almost insignificant exothermic effects. That might be due to the residual air in the materials pores.

As a common technique, thermogravimetric was widely used to evaluate thermal decomposition of biomass or their composite. The FWO and Friedman methods, one of the representative isoconversional methods also were used to determine the kinetic parameters widely. Biagini *et al*.^42^ applied Friedman method to study pyrolysis dynamic of poplar wood using four heating rates of 10, 20, 40, 60 and 80 K min^−1^ by TGA in nitrogen atmosphere and obtained average activation energy of 168.9 kJ mol^−1^. Vecchio *et al*.^43^ investigated the thermal degradation of poplar wood by DSC-TG in air and calculated the kinetic parameters in active pyrolysis region by the Kissinger method and obtained activation energies of 146 kJ mol^−1^ (first peak) and 188 kJ mol^−1^ (second peak). Katarzyna *et al*.^44^ examined thermal degradation of poplar wood by TG/DTG in nitrogen and applied the Kissinger, KAS and FWO methods to calculate the kinetic parameters and obtained activation energies in active pyrolysis region of 153.92, 157.27 and 158.58 kJ mol^−1^, respectively. Sun *et al*.^39^ investigated degradation mechanisms of wood flour/polypropylene composites (WPPC) added the different fire retardants by TG/DTG in a high purity nitrogen stream, and the activation energy values obtained by Kissinger and FWO methods for WPPC with or without fire retardants were 161–178 kJ mol^−1^ (wood flour degradation stage) and 234–305 kJ mol^−1^ (polypropylene degradation stage), respectively.

Currently, some studies for thermostability just used the TG/DTG curves and structure analysis to explain the reason of the thermostability enhanced simply, and not used kinetic models to further analysis the TG/DTG curves^7, 21, 23, 34^. In this work, the pyrolysis process of all samples were fitted by two kinetic models (FWO and Friedman method), which might be able to quantify the TG/DTG curves and improve the accuracy for the experiment. During the thermal degradation of the poplar wood and MnFe_2_O_4_/poplar wood composites under nitrogen and air atmosphere. All of the MPW were in broad agreement with each other in the TG and DTG curves under the same atmosphere, although the maximum weight loss rate temperature in the TG curves for MPW_1_, MPW_2_ and MPW_3_ had shifted to the higher temperature compared the PW. Under the nitrogen atmosphere, activation energy for MPW in whole pyrolysis process obtains a large promotion by coating MnFe_2_O_4_ nanoparticles on the wood surface. Under the air atmosphere, the MnFe_2_O_4_ nanoparticles within MPW exhibited different mechanism for the pyrolysis process that showed a high initial oxidative decomposition temperature. Increasing the proportion of the MnFe_2_O_4_ nanoparticles within the composites could promote activation energy, implying the thermostability of these composites improved as the proportion of the MnFe_2_O_4_ increased. The reason of enhanced thermostability for the poplar wood after coating MnFe_2_O_4_ nanoparticles also could be explained by microscopic view analysis. This study showed the distinctive structure of these composite that the MnFe_2_O_4_ nanoparticles wrapped around the wood particles closely with strong hydrogen bonds association, implying these composites might had a good stability in pyrolysis process.

In addition, the preparation of MnFe_2_O_4_/C composite under the N_2_ atmosphere also showed excellent adsorptive property for methylene blue. The pyrolysis behaviors and kinetic study under N_2_ atmosphere also had played a guiding role for preparation of MnFe_2_O_4_/C composite. For synthesized MnFe_2_O_4_/C composite (heating rate of 20 °C/min), the MnFe_2_O_4_ content of 33.8%, 47.2% and 52.35% could be calculated from residues of MPW_1_, MPW_2_ and MPW_3_ by the TG curves of Fig. 3c, respectively. Due to its high levels of active carbon and easy separation characteristic based on coated MnFe_2_O_4_ nanoparticles, the synthesized MnFe_2_O_4_/C composites might have great potential to be applied in water purification. Thus, a series of adsorption experiments were carried out to estimate the adsorption capacity for methylene blue onto synthesized MnFe_2_O_4_/C composites. As shown in Fig. S1, the MnFe_2_O_4_/C composites displayed a rapid adsorption rate and good adsorption capacity for methylene blue. The inset proved the easy separation characteristic of MnFe_2_O_4_/C composites. Comparison with the activated carbon, the MnFe_2_O_4_/C composites showed a faster adsorption rate, attributing to high affinity between methylene blue and the abundant surface bound hydroxyl radical^45^. Furthermore, although MnFe_2_O_4_ nanoparticle showed an extremely small adsorption capacity (about 3 mg/g) for methylene blue, a markedly enhancement of adsorption capacity of MnFe_2_O_4_/C composite was observed for methylene blue. However, appropriate proportion of MnFe_2_O_4_ for enhancement of adsorption capacity was necessary. An excessive rate of the MnFe_2_O_4_ for composite was able to reduce the adsorption capacity, also indicating that the active carbon in composite played a major adsorption for methylene blue. In this work, the maximum adsorption capacity (adsorption centage) of CPW, CMPW_1_, CMPW_2_ and CMPW_3_ are close to 81.71 mg/g (81.71%), 84.18 mg/g (84.18%), 74.44 mg/g (74.44%), and 72.53 mg/g (72.53%), respectively. And the maximum adsorption capacity for methylene blue of the MnFe_2_O_4_/C composite was higher than some previously reported adsorption capacity, such as PAA/MnFe_2_O_4_ nanocomposite (53.3 mg/g)^46^, magnetic graphene-carbon nanotube composite (65.79 mg/g)^47^ and CNT (45.9 mg/g)^48^. Thus, these composites had high application potential used to remove methylene blue in aqueous solution due to its high adsorption capacity and easy separation characteristic.

## Conclusions

In this work, we studied the thermostability for MnFe_2_O_4_/poplar wood composites in the N_2_ and air atmosphere by TG/DTG and DSC analysis. The MnFe_2_O_4_/poplar wood composite showed high active energies in N_2_ atmosphere, indicating its good thermostability. And the high initial oxidative decomposition temperature in air atmosphere implied wide applications for this composite and its preparation method, such as flameresistant material and security storage of wood. The method also could provide a reference for other biomass materials via a simple and low temperature hydrothermal reaction to coat MnFe_2_O_4_ nanoparticles on materials surface. In addition, under the guidance of pyrolysis behaviors and kinetic study in N_2_ atmosphere, the MnFe_2_O_4_/C composite was successfully synthesized and exhibited good adsorption capacity for removing methylene blue dye in aqueous solution and easy separation characteristic due to its excellent magnetic responsiveness from MnFe_2_O_4_ nanoparticles.

## Materials and Methods

### Materials

The poplar wood samples (*Populus Alba)* were collected from fast grown poplar in Zhejiang A&F University. Before the experiments, the poplar wood samples were processed into the powder with a size of 200 meshes, and dried at 105 °C for 12 h in a vacuum. Then, the poplar wood powder was stored in the vacuum desiccators. In addition, all chemicals in this work were supplied by Sigma-Aldrich and used without further purification.

### Synthesis of MnFe_2_O_4_/poplar wood composite (MPW) via hydrothermal method

MnSO_4_·H_2_O (1.352 g) and FeCl_3_·6H_2_O (4.324 g) in a stoichiometric ratio of 1:2 were dissolved in 40 mL of deionized water under magnetic stirring at room temperature. The obtained homogeneous mixture with different dosages and 0.5 g of poplar wood powder (PW) were transferred into five Teflon-lined stainless autoclave (Standard: 50 mL) and labeled these as PW (0 mL), MPW_1_ (2 mL), MPW_2_ (4 mL) and MPW_3_ (5 mL), respectively. Then, 0 mL, 1 mL, 2 mL and 2.5 mL of ammonia were separately added into the PW, MPW_1_, MPW_2_ and MPW_3_. And all of these were diluted with water to 40 mL. After that, the Teflon-lined stainless-steel autoclave was sealed and heated to 110 °C for 6 hours. Subsequently, the autoclaves were left to cool down to room temperature (25 °C). Finally, the prepared MnFe_2_O_4_/poplar wood composite (MPW) were removed from the solution in the filters with deionized water and alcohol, and dried at 45 °C for over 24 hours in vacuum. All samples were stored in the vacuum desiccators for experiment. As a control, pure MnFe_2_O_4_ nanoparticles were prepared under identical conditions. The percentage of theoretical contents of MnFe_2_O_4_ for MPW_1_, MPW_2_ and MPW_3_ were 15.58%, 26.96% and 31.57% calculated from ratio and mass of raw materials, respectively.

### Characterizations

Proximate analysis of the PW was performed according to Chinese National Standards GB/T28731-2012. The elemental analyses of carbon, hydrogen, nitrogen and sulfur of the dried PW feedstock were determined by the CHNS/O model using an elemental analyzer (Vario EL III, Elementary, Germany), and the oxygen content was then calculated by difference. Lower heating value (LHV) of PW was calculated by automatic calorimeter (ZDHW-300A, Hebi Keda Instrument & Meters Co., LTD, China). These results were listed in Table 2.

**Table 2:** Elemental, proximate and biochemical analysis of the PW.

Elemental analysis, ash free (mass %)		Proximate analysis, dry basis (mass %)		Biochemical analysis (mass %)	
Carbon	50.43	Volatiles	75.11	Cellulose	48.26
Oxygen	42.18	Fixed carbon	23.21	Hemicellulose	19.39
Hydrogen	6.45	Ash	1.68	Lignin	29.90
Nitrogen	0.61	LHV/MJ·Kg^−1^	18.38		
Sulfur	0.33				

Crystalline structures of the samples were identified by the X-ray diffraction technique (XRD, Rigaku, D/MAX 2200) operating with Cu Kα radiation (λ = 1.5418 Å) at a scan rate (2θ) of 4° min^−1^ and the accelerating voltage of 40 kV and the applied current of 30 mA ranging from 10° to 70°. The functional groups were recorded via a Fourier transform infrared spectroscopy (FT-IR, Magna-IR 560, Nicolet). The spectra were obtained at a resolution of 4 cm^−1^ in the range from 400 to 4000 cm^−1^. The morphologies of the samples were characterized by the transmission electron microscope (TEM, FEI, Tecnai G20). The functional groups and elemental composition were conducted on X-ray photoelectron spectrometer (ESCALAB 250 XI, Thermofisher Co.). The thermogravimetric decomposition experiments were performed using a thermogravimetric analyzer (Netzsch, TG209F1, Germany) with a readability of 0.1 micrograms. The thermogravimetric(TG)/differential thermogravimetric(DTG) and differential scanning calorimetry (DSC) test was fulfilled at the N_2_ atmosphere with a flow rate of 40 mL/min and air atmosphere. The heating rate were 10 °C/min, 20 °C/min, 30 °C/min and 40 °C/min for PW, MPW_1_, MPW_2_ and MPW_3_ samples. The TG/DTG curves were applied to obtain the key pyrolysis parameters for samples, including maximum weight loss rate r_m_ (wt. %/min), initial temperature T_0_ (°C), pyrolysis temperature T_p_ (°C), temperature at maximum weight loss rate T_m_ (°C) and total mass loss for pyrolyzation (wt.%).

### Non-isothermal kinetic model on Kinetic study

The thermal decomposition of PW and MPW were investigated by the kinetic scheme, and the kinetic model of pyrolysis could be established based on the TG/DTG curve obtained from the thermogravimetric analysis. In this work, Arrhenius equation was selected to analyze pyrolysis process of the samples. In the gas-solid reaction, the non-isothermal thermal reaction rate equation was shown by Eqs (1–3):

1 $$\frac{d\alpha }{dt}=A\exp (-\frac{E}{RT})f(\alpha )$$

2 $${\rm{\alpha }}=\frac{{m}_{0}-{m}_{t}}{{m}_{0}-{m}_{f}}$$

3 $${\rm{\beta }}=\frac{dT}{dt}$$

where the α is the degree of conversion, t is the reaction time, A is the pre-exponential factor of the pyrolysis, E is the activation energy, R is the universal gas constant (8.314 J/(mol·K)), T is the temperature in Kelvin (K), *f*(*α*) is the differential mechanism function, m_0_ is initial sample weight, m_t_ is sample weight at time t, m_f_ is final sample weight and the heating rate β is defined as Eq. (3).

Substituting Eq. (3) into the Eq. (1), as follows:

4 $$\frac{d\alpha }{dT}=\frac{A}{\beta }\exp (-\frac{E}{RT})f(\alpha )$$

5 $${\rm{G}}({\rm{\alpha }})={\int }_{0}^{\alpha }\frac{d\alpha }{f(\alpha )}=\frac{A}{\beta }{\int }_{0}^{T}\exp \,(-\frac{E}{RT})dT=\frac{AE}{\beta R}P(\mu )$$

where G(a) is the integral form of *f*(*α*), P(u) is an approximation, and u is defined as the equation of u = E/R.

### Model-free method

In order to obtain the value of activation energy (E), the Flynn Wall Ozawa (FWO) method and Friedman method were used as a model of free integral method in this work. The estimated activation energy is a function of the conversion rate (α) in this method. To obtain reliable values of the activation energy, four heating rates (10, 20, 30 and 40 °C/min) were used in the thermogravimetric analysis. The equation of FWO and Friedman were defined at Eqs 6 and 7, as follow:

6 $$\mathrm{lg}\,\beta =\,\mathrm{lg}\,\frac{AE}{RG(\alpha )}-2.315-0.4567\frac{E}{RT}$$

7 $${\rm{In}}(\frac{d\alpha }{dt})={\rm{InA}}f(\alpha )-\frac{E}{RT}$$

The linear plots of lgβ vs. 1/T and In (dα/dt) vs. 1/T could reach value of activation energy (E) corresponding to each conversion rate (α). Observably, the $\mathrm{lg}(\frac{{\rm{AE}}}{{\rm{RG}}(\alpha )})-2.315$ and −0.4567*E*/*R* by FWO method and InA*f*(*α*) and –*E*/*R* by Friedman method in the linear plots were corresponding to the intercept and slope, respectively. Generally, four heating rates (10, 20, 30 and 40 °C/min) could obtain accurate and reliable values for the activation energy.

### Batch adsorption experiment for methylene blue dye

The MnFe_2_O_4_/C composite for CMPW_X_ (X = 1, 2, 3, and 4) and wood charcoal for CPW were prepared by pyrolysis of the MnFe_2_O_4_/wood composite and poplar wood at the heating rate of 20 °C/min under N_2_ atmosphere, respectively. Batch adsorption experiments were carried out at room temperature to estimate the adsorption capacity for methylene blue of MnFe_2_O_4_/C composite, wood charcoal and pure MnFe_2_O_4_ nanoparticles, where the adsorbents of 15 mg were added to 100 mL methylene blue solutions (15 mg/L). Then, the UV–vis spectrophotometer (Purkinje TU-1901) was applied to analyze methylene blue dye concentration at their corresponding maxima wavelength. And the absorbance for all solution was obtained at certain time intervals during the adsorption process.

## Electronic supplementary material

Supplementary Information
