# Supplementary material for: New Insight on Promoted thermostability of poplar wood modified by MnFe2O4 nanoparticles through the pyrolysis behaviors and kinetic study
Source: Sci Rep. 2017 May 3;7:1418. doi: 10.1038/s41598-017-01597-4 (PMC5431171; doi:10.1038/s41598-017-01597-4)
Supplement: Supplementary file 1 — Supplementary Information [file 41598_2017_1597_MOESM1_ESM.pdf]

New Insight on Promoted thermostability of poplar wood modified by $MnFe_2O_4$ nanoparticles through the pyrolysis behaviors and kinetic study

Hanwei Wang[1], Qiufang Yao[1], Chao Wang[1], Bitao Fan[1], Ye Xiong[1], Yipeng Chen[1], Qingfeng Sun[1, 2*], Chunde Jin[1, 2*], Zhongqing Ma[1, 2*]

[1]School of Engineering, Zhejiang A&F University, Lin'an, China

[2]Key Laboratory of Wood Science and Technology, Lin'an, China

[*]Corresponding author: qfsun@zafu.edu.cn (Qingfeng Sun), jincd@zafu.edu.cn (Chunde Jin), & mazqzafu@163.com (Zhongqing Ma)

Postal address: School of Engineering, Zhejiang A&F University, Huancheng North Road NO.88, Lin’an City, Zhejiang Province, 311300, P. R. China

Tel: +86- 571- 63732601; Fax: +86-571- 63732601

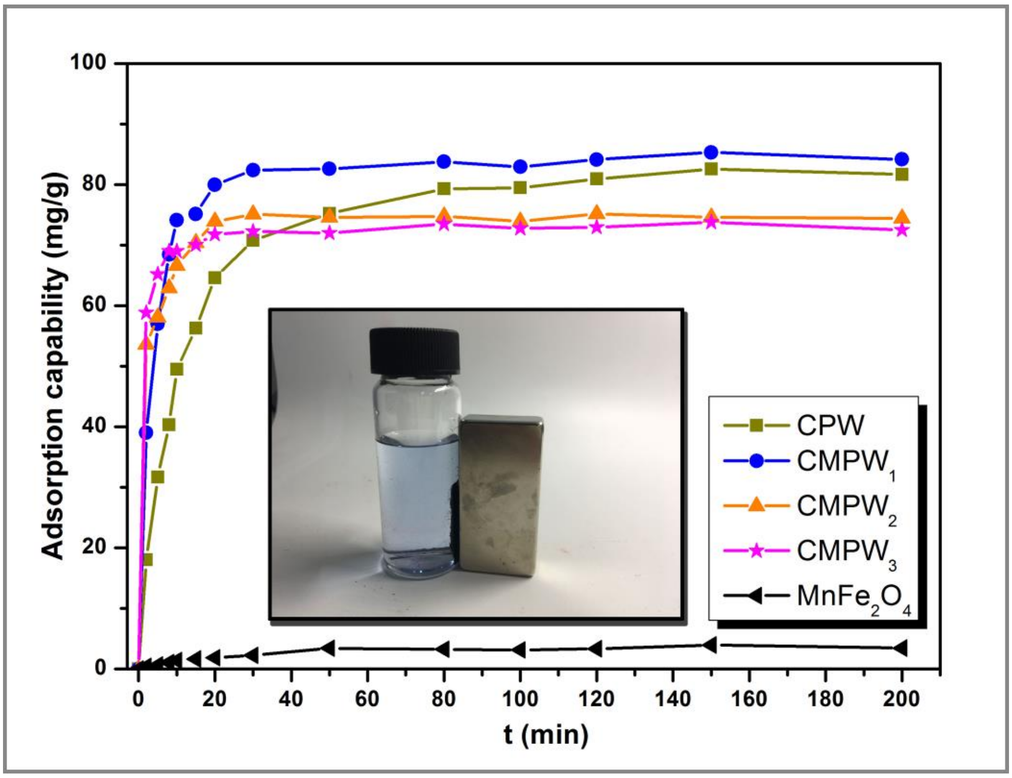

Fig. S1 Adsorption capability of methylene blue onto the synthesized CPW and CMPW. The inset showed easy separation characteristic for CMPW. (Experimental conditions: C_0 = 15 mg/L, samples dosage = 150 mg/L, T = 25 ◦C).

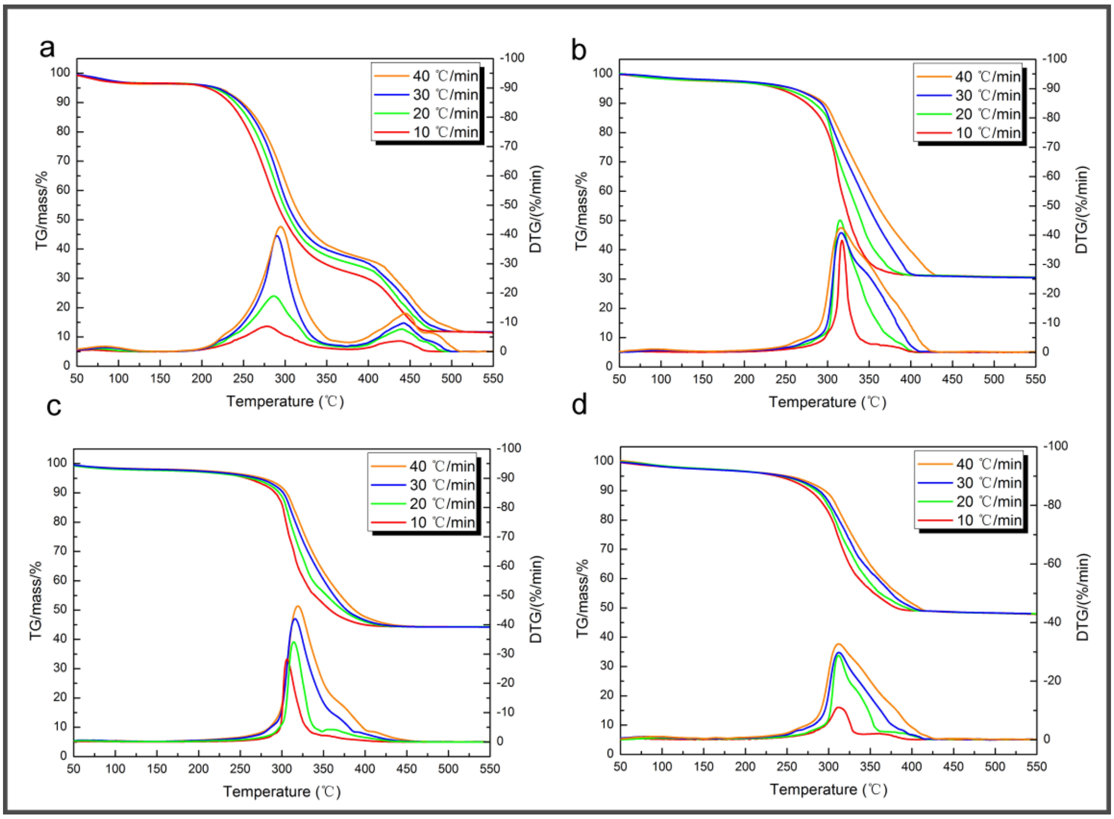

Fig. S2 TG and DTG curves of (a) PW, (b) MPW_1, (c) MPW_2 and (d) MPW_3 with heating rates of 10, 20, 30 and 40 ℃/min in air atmosphere.

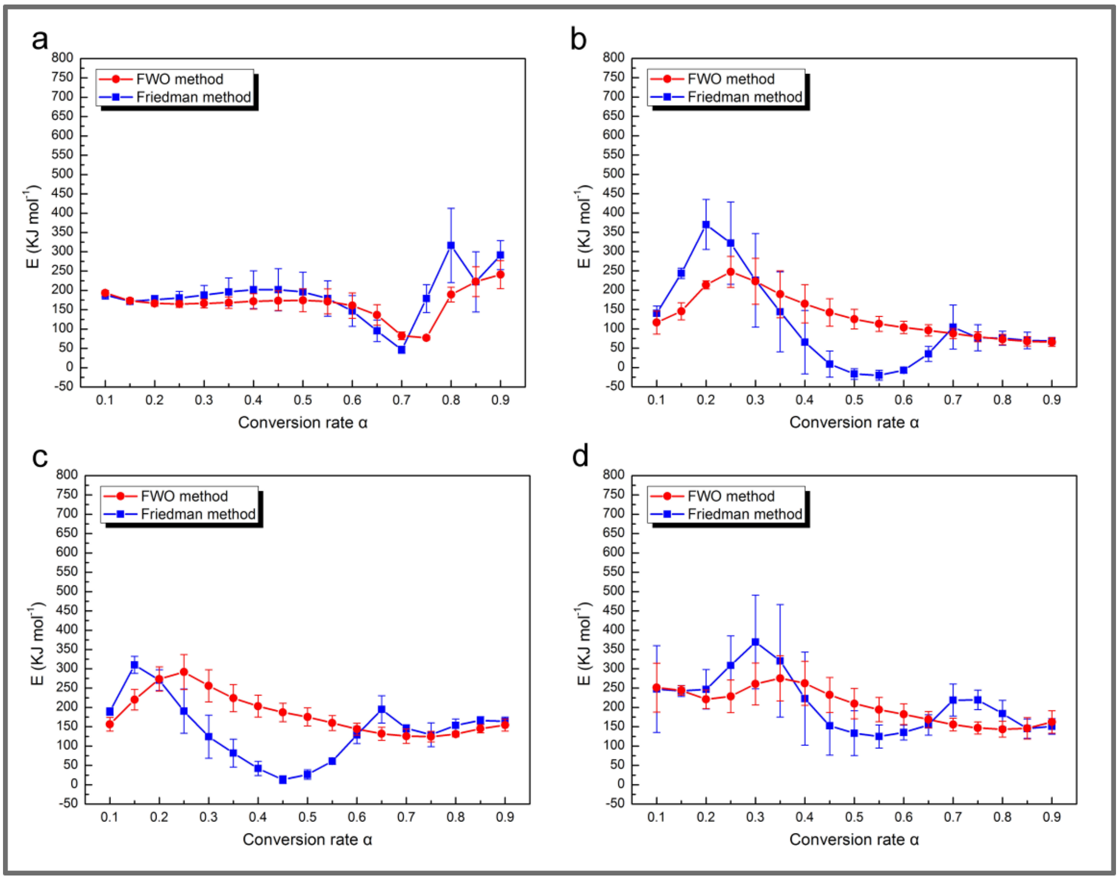

Fig. S3 Activation energy (E) distribution with corresponding error bar in the different conversion rates using the FWO and Friedman methods for (a) PW, (b) MPW_1, (c) MPW_2 and (d) MPW_3 in air atmosphere.

Table S1

TGA pyrolysis of PW, MPW_1, MPW_2 and MPW_3 activation energies (E) and standard deviation (error bar) for different conversion values using the FWO and Friedman models in nitrogen atmosphere.

Conversion (a)	Activation energy FWO model (kJ/mol)	Standard Deviation (±kJ/mol)	Activation energy Friedman model (kJ/mol)	Standard Deviation (±kJ/mol)
PW				
0.10	143	30	148	29
0.15	150	24	143	30
0.20	151	23	149	22
0.25	154	20	148	17
0.30	154	18	147	14
0.35	155	16	149	15
0.40	156	16	154	17
0.45	157	16	155	16
0.50	159	15	156	16
0.55	160	15	158	17
0.60	161	15	160	17
0.65	163	15	164	18
0.70	166	16	175	22
0.75	175	20	205	36

0.80	206	36	249	54
0.85	225	53	216	56
0.90	225	81	224	89
MPW_1				
0.10	177	58	258	35
0.15	223	34	253	46
0.20	227	28	227	33
0.25	224	25	226	30
0.30	221	23	227	20
0.35	221	20	226	14
0.40	215	16	212	11
0.45	213	15	207	10
0.50	212	13	216	10
0.55	212	13	221	13
0.60	213	12	221	11
0.65	214	12	225	12
0.70	216	12	234	13
0.75	223	13	253	16
0.80	242	19	309	39
0.85	326	92	382	135
0.90	366	67	442	46
MPW_2				

0.10	280	133	269	101
0.15	276	46	298	17
0.20	282	9	318	34
0.25	256	9	245	5
0.30	244	2	232	22
0.35	234	6	229	8
0.40	229	5	225	7
0.45	245	6	221	8
0.50	221	6	220	8
0.55	219	6	220	7
0.60	218	6	221	8
0.65	218	6	229	7
0.70	220	6	235	6
0.75	224	7	253	9
0.80	247	2	318	6
0.85	338	37	385	46
0.90	386	45	430	57
MPW_3				
0.10	292	125	270	102
0.15	262	50	313	26
0.20	270	30	302	56
0.25	247	31	236	35

0.30	235	23	229	36
0.35	227	20	218	18
0.40	217	17	203	13
0.45	213	15	204	11
0.50	210	13	210	10
0.55	208	13	213	11
0.60	209	12	218	12
0.65	209	12	226	11
0.70	218	13	256	17
0.75	252	18	322	22
0.80	291	32	318	37
0.85	322	33	346	31
0.90	425	46	479	51

Table S2

Effect of heating rate on TGA pyrolysis for the PW, MPW_1, MPW_2 and MPW_3 in air atmosphere.

Sample	β (℃/min)	r_m (wt.%/min^{-1})	T_m (℃)	Residue (%dry)
PW	10	-8.65	278.34	11.24
	20	-18.92	287.44	11.68
	30	-39.56	290.24	11.70
	40	-42.64	294.54	11.71
MPW_1	10	-38.13	307.30	30.61
	20	-45.11	314.93	30.77
	30	-40.81	316.30	30.89
	40	-42.43	317.35	30.67
MPW_2	10	-28.24	306.28	44.25
	20	-34.12	314.32	44.31
	30	-42.03	315.47	44.34
	40	-46.35	319.15	44.29
MPW_3	10	-11.07	311.75	48.20
	20	-28.74	311.80	48.25
	30	-29.77	312.18	48.30
	40	-32.69	312.39	48.32

Table S3

TGA pyrolysis of PW, MPW_1, MPW_2 and MPW_3 activation energies (E) and standard deviation (error bar) for different conversion values using the FWO and Friedman models in air atmosphere.

Conversion (a)	Activation energy FWO model (kJ/mol)	Standard Deviation (±kJ/mol)	Activation energy Friedman model (kJ/mol)	Standard Deviation (±kJ/mol)
PW				
0.1	143	30	148	29
0.15	150	24	143	30
0.2	151	23	150	22
0.25	154	20	148	17
0.3	154	18	147	14
0.35	155	16	149	15
0.4	156	16	154	17
0.45	157	16	155	16
0.5	159	15	156	16
0.55	160	15	158	17
0.6	161	15	160	17
0.65	163	15	164	18
0.7	166	16	175	22
0.75	175	20	205	36
0.8	206	36	249	54
0.85	225	53	216	56
0.9	224	81	224	89
MPW_1				
0.1	117	30	140	19
0.15	146	22	244	13
0.2	214	10	370	65

	0.25	248	40	322	107
	0.3	223	60	226	121
	0.35	190	61	144	104
	0.4	165	50	66	82
	0.45	143	36	9	34
	0.5	125	25	-17	14
	0.55	113	19	-20	13
	0.6	104	16	-7	8
	0.65	96	14	35	19
	0.7	88	13	104	57
	0.75	80	13	77	34
	0.8	73	13	76	18
	0.85	68	12	70	21
	0.9	66	11	69	9
MPW_2					
	0.1	156	18	190	10
	0.15	220	27	310	22
	0.2	274	32	271	27
	0.25	292	45	190	57
	0.3	256	42	124	56
	0.35	224	35	82	36
	0.4	203	28	42	19
	0.45	187	24	13	10
	0.5	176	23	26	12
	0.55	160	19	61	9
	0.6	144	15	129	22
	0.65	132	17	195	35
	0.7	126	19	146	9
	0.75	124	14	129	31
	0.8	131	7	153	17
	0.85	145	11	167	9
	0.9	154	16	164	11
MPW_3					

0.1	251	63	248	113
0.15	244	12	246	14
0.2	221	24	247	51
0.25	229	42	309	77
0.3	261	54	370	121
0.35	276	59	321	146
0.4	263	57	223	121
0.45	232	45	152	76
0.5	210	39	133	58
0.55	194	31	125	30
0.6	182	27	135	19
0.65	169	21	155	26
0.7	156	16	219	42
0.75	147	16	220	25
0.8	144	20	184	34
0.85	146	28	145	25
0.9	162	29	151	20
